# The Role of the Blood-Brain Barrier in the Pathogenesis of Senile Plaques in Alzheimer's Disease

**DOI:** 10.1155/2014/191863

**Published:** 2014-09-18

**Authors:** J. Provias, B. Jeynes

**Affiliations:** ^1^Department of Pathology & Molecular Medicine (Neuropathology), Hamilton Health Sciences, McMaster University, 1280 Main Street West, Hamilton, ON, Canada L8S 4L8; ^2^Department of Health Sciences, Faculty of Applied Health Sciences, Brock University, 500 Glenridge Avenue, STH 315, St. Catharines, ON, Canada L2S 3A1

## Abstract

The accumulation of beta-amyloid [A*β*] within senile plaques [SP] is characteristic of these lesions in Alzheimer's disease. The accumulation of A*β*_42_, in particular, in the superior temporal [ST] cortex may result from an inability of the blood brain barrier (BBB) to regulate the trans-endothelial transport and clearance of the amyloid. Lipoprotein receptor-related protein [LRP] and P-glycoprotein [P-gp] facilitate the efflux of A*β* out of the brain, whereas receptor for advanced glycation end products [RAGE] facilitates A*β* influx. Additionally, vascular endothelial growth factor [VEGF] and endothelial nitric oxide synthase [eNOS] may influence the trans-BBB transport of A*β*. In this study we examined ST samples and compared SP burden of all types with the capillary expression of LRP, p-gp, RAGE, VEGF, and e-NOS in samples from 15 control and 15 Alzheimer brains. LRP, P-gp, RAGE, VEGF, and eNOS positive capillaries and A*β*_42_ plaques were quantified and statistical analysis of the nonparametric data was performed using the Mann-Whitney and Kruskal-Wallis tests. In the Alzheimer condition P-gp, VEGF, and eNOS positive capillaries were negatively correlated with SP burden, but LRP and RAGE were positively correlated with SP burden. These results indicate altered BBB function in the pathogenesis of SPs in Alzheimer brains.

## 1. Introduction

Although the pathogenesis of Alzheimer's disease, including the pathophysiology leading to excessive A*β*_42_ deposition in AD brain, remains unknown, it is characterized by the excessive accumulation of beta-amyloid peptides in varying size aggregates, predominantly within neocortex [1–3]. This study looks at one of the characteristic areas of cortical involvement, the superior temporal region, to examine more specifically the relationship between the degree of A*β*_42_ peptide accumulation in the form of plaques, with various indicators of vascular endothelial function and beta-amyloid peptide transport.

Research has focused largely on the amyloid cascade theory predicated upon increased production of beta amyloid from amyloid precursor protein precursors as a function of altered processing [3]. Alternatively, impaired transport and elimination of beta amyloid from CNS parenchyma into the CSF and vascular compartments may be a significant contributor and/or the dominant mechanism leading to this accumulation. Evidence for this comes from a number of fronts, including the strong association and overlap of Alzheimer's disease with vascular comorbidities [4].

Indeed it has been suggested by some that Alzheimer's disease is a form of vascular dementia [4–6]. There is a strong association with specific cardiovascular disease risk factors, such as hyperlipidemia, elevated homocystein levels, obesity, diabetes, cardiac disease, apo-E alleles, hypertension and strokes, and others.

At a global level within the brain, the development of vascular disease associated with aging and these risk factors leading to vascular fibrosis and increased rigidity has been shown to be associated with impaired interstitial perivascular drainage, elimination, and beta-amyloid efflux [4, 7]. At the microvascular level, dysfunction of endothelial cells and blood-brain barrier can be associated with reduced cerebral blood flow and hypoxia. Further, A*β* deposition itself within microvessels can contribute to further endothelial cell damage [8, 9].

Thus, vascular dysfunction and microvessel/endothelial blood-brain barrier dysfunction are important parameters to be evaluated in understanding the potential pathophysiology of beta-amyloid accumulations and plaque formation in AD cortex. This study looks at that relationship, specifically correlating A*β*_42_ senile plaque levels with proteins known to play a role in microvascular/endothelial blood-brain barrier function, that is, eNOS and vascular endothelial growth factor (VEGF) and specific transport proteins for beta amyloid which exist at the blood-brain barrier; LRP, P-glycoprotein, and RAGE.

Under normal physiologic conditions it is presumed that the load of beta amyloid within the cerebral interstitium and by extension cerebral parenchyma is in healthy individuals regulated by interstitial production control and by regulation at the blood-brain barrier of efflux out of the brain into the vascular and cerebrospinal fluid compartments by LRP and P-glycoprotein [10]. This is balanced by influx from the vascular compartment into the cerebral interstitium regulated by RAGE activity [11]. Thus vascular structural integrity, especially at the level of microvessels of blood-brain barrier, as well as the overall capillary/blood-brain barrier capacity for homeostatic function is critical in balancing this influx/efflux equation and hence normal physiologic levels of beta-amyloid peptides. Imbalance in this relationship putatively contributes to beta-amyloid accumulation and plaque pathogenesis.

In addition to evidence implicating vascular disease and cardiovascular risk factors in AD development, vascular endothelial growth factor itself has been shown to be altered in Alzheimer's disease. Increased VEGF has been described in CSF while serum levels of VEGF have been shown to be reduced [12, 13]. In addition, microvessels isolated from AD brain have been shown to have elevated VEGF secretion and increased VEGF responsiveness to oxidative stress [14]. In transgenic mouse models, increased VEGF expression has been shown to lead to improvement of learning behaviour. VEGF has also been shown to be altered and probably increased in brain parenchyma, in association with the development of reactive astrocytic gliosis [12]. Furthermore, VEGF, in addition to its well-known angiogenic and endothelial stimulating properties, has been shown to have a neuronal protective effect, so altered VEGF levels may contribute more directly to neuronal degeneration [13].

The sources of A*β* accumulated in senile plaques are known to be multiple. The function of *β*-amyloid peptide remains obscure. It is found naturally within circulating plasma, being generated, for example, in the liver and is known to be produced by neuronal and cerebral astrocytes [15]. Critically for lesion development, the load of amyloid in the cerebral interstitium is in healthy individuals regulated by interstitial production control and by the influx/efflux regulation in and out of the brain described above. Imbalance by either process may contribute to senile plaque pathogenesis. Additionally, it has been demonstrated that breaches of the endothelial tight junctions of the blood-brain barrier are likely associated with a number of neuropathologies, including Alzheimer's disease [4, 8, 16].

In this study we analyse quantitative results in order to more comprehensively compare the relationship between A*β*_42_ plaques and each of the *β*-amyloid transport and vasoactive proteins.

## 2. Methods

15 cases of Alzheimer's disease and 15 cases of control non-Alzheimer, nondemented, and nonneurologic causes were selected from the archival autopsy material of the HHS (Hamilton Health Sciences). Alzheimer's disease (AD) cases were assessed utilizing standard neuropathologic criteria including H. Braak and E. Braak [17] staging and CERAD [18] probability indexing. All 15 AD cases were free from any significant other degenerative confounding neuropathologies and from significant intracranial vascular pathology or infarcts. Tissue blocks were paraffin processed. Sections were routinely stained with Luxol Fast Blue/Hematoxylin and Eosin. Routine diagnostic assessment included immunohistochemistry for tau (Dako, rabbit polyclonal, C-terminus specific), beta amyloid (Dako, mouse monoclonal N-terminus_8–17_), ubiquitin (Dako, rabbit polyclonal), and alpha-synuclein (Zymed, mouse monoclonal). Superior temporal blocks underwent further immunohistochemical assessment with antibodies to beta amyloid_1–42_ (ID Labs Inc., rabbit polyclonal), eNOS (Nova Castra, mouse monoclonal) and VEGF (N-terminus specific, ID Labs, rabbit polyclonal), LRP-1 (Biodesign International ME (USA), mouse monoclonal), RAGE (Northwest Life Science Specialties, rabbit polyclonal), and P-gp (Abcam, Cambridge, MA, mouse monoclonal (C494)). All the immunohistochemistry was performed on standard 7 *μ*m tissue sections using Avidin Biotin Complex (ABC) methodology with hematoxylin nuclear counterstaining. AD cases were diagnosed utilizing standard criteria based on the presence of beta-amyloid positive plaques and neuronal neurofibrillary tau degeneration. In all of the selected cases, areas of superior temporal cortices were selected for quantitative analysis of beta-amyloid_42_ [A*β*_42_] plaques and for positive capillary LRP, RAGE. P-gp, ENOS, and VEGF expression. For each region, a start point was randomly determined and 10 contiguous fields were examined. All fields were examined at 200x magnification. In this manner, A*β*_42_ senile plaques and LRP, RAGE, P-gp, eNOS, and VEGF positive capillaries were counted and total counts, as well as densities (counts per field area), were determined for each. All A*β*_42_ plaques (SPs) were counted, including diffuse, neuritic, and core forms.

LRP, RAGE. P-gp, ENOS, and VEGF positive capillaries were counted, with capillaries being defined as vessels less than 10 *μ*m in diameter and morphologically consisting of only an endothelium and basal lamina.

Statistical analysis of the data was performed using the nonparametric data Mann-Whitney Rank Sum and Kruskal-Wallis tests for comparison of Alzheimer and control group conditions and sites. The Spearman's nonparametric correlation test was used to correlate the different lesion data within a specific region and condition.

## 3. Results

This study compares the senile plaque burden in control and Alzheimer brains within the superior temporal cortex with the expression of blood-brain barrier endothelial proteins associated with the transfer and transport of amyloid (Table 1).

**Figure 1:**
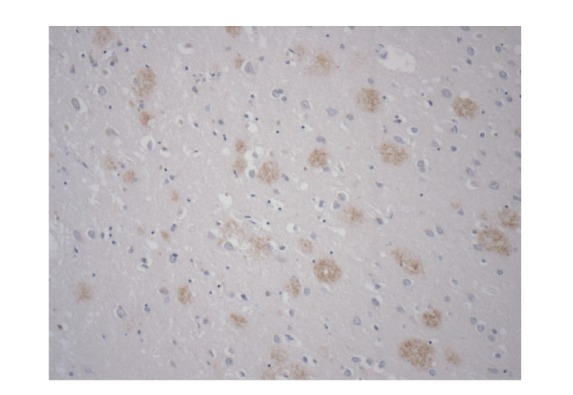


Figure 1 illustrates immunohistochemically stained A*β*_42_ senile plaques (×100).

**Figure 2:**
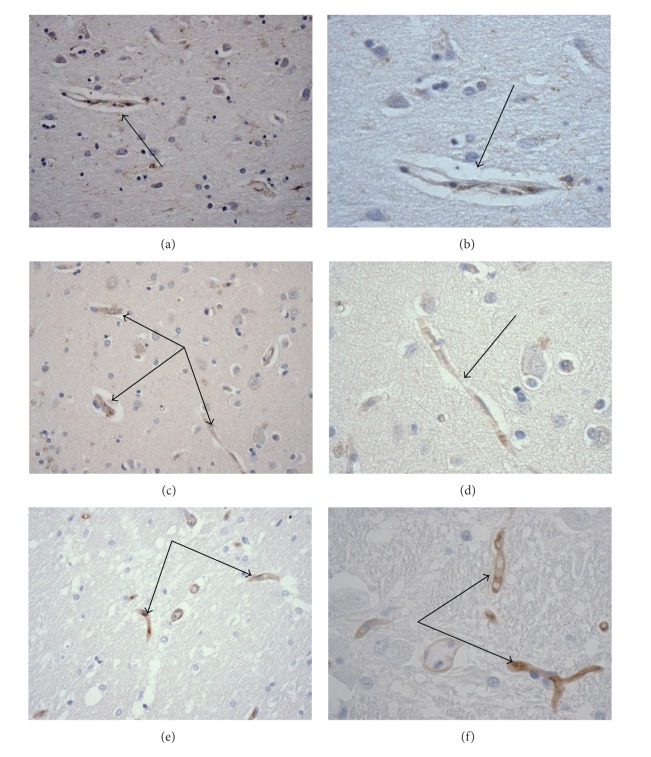
Immunohistochemical illustrations (arrows) of blood-brain barrier endothelial cell associated amyloid transport proteins. Immunohistochemical staining for (a and b) LRP, ×200 & ×400, respectively; (c and d) RAGE, ×200 & ×400, respectively; (e and f) P-gp, ×200 & ×400, respectively.

Figure 2 illustrates immunohistochemically stained LRP, RAGE, and P-gp blood-brain barrier associated endothelial cells in cortical regions of AD brains.

**Figure 3:**
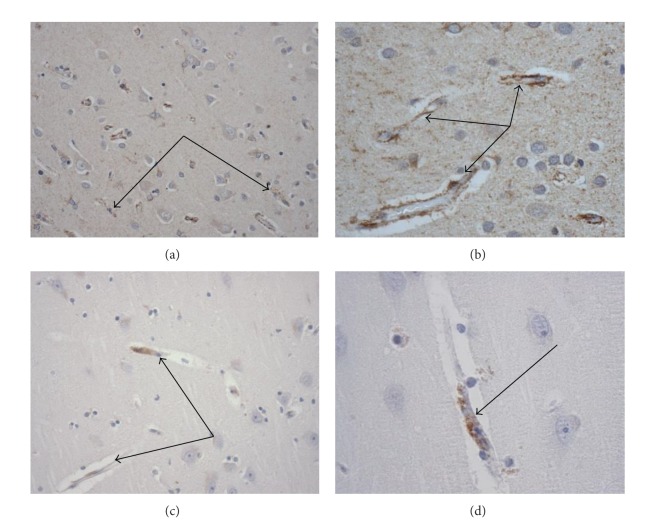
Immunohistochemical illustrations (arrows) of blood-brain barrier endothelial cell associated and vasoactive proteins. Immunohistochemical staining for (a and b) VEGF, ×200 & ×400, respectively; (c and d) eNOS, ×200 & ×400, respectively.

Figure 3 illustrates immunohistochemically stained VEGF and eNOS blood-brain barrier associated endothelial cells in cortical regions of AD brains.

The raw data represents the total counts for 10 fields from the superior temporal cortex in each condition. It indicates a clear significant difference between senile plaque burden comparing control and Alzheimer conditions. With respect to the raw data for the endothelial protein expressions, though there appear to be differences between the two conditions for each protein, there are only significant differences between the two conditions for RAGE and VEGF expression (Table 2).

When correlating the associations between A*β*_42_ plaque burden and the endothelial expression for each of the proteins, there were significant positive A*β*_42_ plaque correlations with eNOS and VEGF positive capillary expression in the control condition but negative correlations for both in the Alzheimer condition. With respect to A*β*_42_ plaque correlations with positive capillary LRP, RAGE, and P-gp expression, there were no significant correlations in the control condition. In the Alzheimer condition there were, however, positive correlations between A*β*_42_ plaque burden and positive LRP and RAGE capillary expression and a significant negative A*β*_42_ plaque burden correlation with positive P-gp capillary expression (Table 3).

## 4. Discussion

In our view, it is important to examine the involvement of trans blood-brain barrier transport proteins [19] and vasoactive proteins in regulating the levels of amyloid accumulation separately, as the mechanisms by which they influence plaque pathogenesis are likely very different.

Considerable effort was expended in obtaining a meaningful control group. In particular, cases were selected to be free of significant neuropathology. Ideally, the control group should be age-matched. Our control group was only partially age-matched because of the availability of control brains, particularly those that met our criteria. Practical realities with regard to the availability of brains in this time of declining autopsy rates create difficult harvesting challenges. However, we have reason to believe that the inferences drawn from the results of this study remain valid in spite of this concern.

The vasoactive proteins eNOS and VEGF are known to influence vascular permeability [5] and therefore may participate in the influx and accumulation of vascular amyloid. VEGF has been well established as an endothelial cell angiogenic factor and mitogen as well as blood-brain barrier permeability regulator [7]. Reduced VEGF levels within this microvascular fraction of the brain by impairing overall angiogenesis and possibly reducing blood-brain barrier permeability could alter the normal homeostatic balance of beta-amyloid peptide handling, leading to impaired efflux out of the CNS parenchymal compartment leading to net accumulation.

This study has shown a significant inverse correlation between beta-amyloid plaque burden and microvessel or capillary expression of both eNOS and VEGF in the superior temporal cortex of Alzheimer brains. This in itself does not establish any causality but does implicate further the role of these proteins in AD pathogenesis potentially by contributing in some fashion to the net beta-amyloid peptide accumulation in the CNS. In transgenic mice models increasing VEGF expression leads to improved learning behaviour, although it is not clear if this is related to reduced beta-amyloid burden or some more direct effect [20]. It remains unknown whether or not microvascular impairment is a primary abnormality leading to reduced VEGF levels and consequent increased beta-amyloid levels or whether increased perivascular parenchymal beta-amyloid peptide leads to microvascular/endothelial cell toxicity and secondary reduction in endothelial VEGF levels [7, 21].

The reduction in expression of eNOS and its inverse correlation to the beta-amyloid plaque burden are significant in that this would lead to altered vasodilation with net reduction in cerebral blood flow, leading to further impairment of net beta-amyloid efflux via the parenchyma to vascular route [7, 19, 22]. Together the similar significant reduction of both of these angiogenic vasoregulatory factors significantly correlated with the beta-amyloid peptide plaque burden strengthens and further points to vascular, and specifically microvascular, dysfunction as being significant and potentially a contributory pathophysiologic factor in AD development.

The trans-endothelial transport by LRP, P-gp, and RAGE is dependent on their role as active carrier molecules of amyloid [19]. LRP and P-gp are known to be the significant efflux transporters of A*β* from the brain. LRP is part of a large family of transmembrane signalling receptor proteins. They are multifunctional and interact with numerous naturally occurring ligands, one of which is beta amyloid. It is known that LRP is expressed in the central nervous system and has been shown to be highly enriched in neuronal cells and expressed by glia and vascular endothelial and smooth muscle cells [23–25]. LRP expression by vascular endothelial cells and its binding to beta amyloid allow it to play a potentially significant role in the beta-amyloid movement from the CNS parenchyma compartment to the vascular compartment and enhance ultimate clearance [23]. A number of lines of evidence have previously implicated LRP in AD pathogenesis. Genetic studies have recently shown a linkage between an LRP polymorphism (C667T) and AD [26]. It has also been shown that increasing the liver expression of LRP is able to reverse CNS AD pathology, presumably by enhancing peripheral A-beta clearance [25]. In vitro studies have confirmed that LRP mediates the beta-40/42 peptide clearance through a true receptor signalling mechanism. As well, utilizing an antisense oligonucleotide strategy, knockdown of LRP-1 in mice was shown to significantly reduce A-beta clearance, leading to increased brain A-beta, AD pathology, and impaired cognitive behavior [27, 28]. It is interesting to note that complete LRP knockout is embryo lethal. The current study while showing no significant difference in overall capillary expression between the two conditions did show a positive correlation with *β* amyloid plaque burden pointing towards a functional role in AD. The mechanistic relationship remains unknown and is not answered directly by this study; however it is possible that LRP is upregulated in response to the increased *β* amyloid burden in an unsuccessful attempt to maintain homeostatic clearance.

P-glycoprotein is part of the ABC family of multidrug efflux transporter proteins (ATP binding cassette family). In the central nervous system it is normally expressed on brain vascular endothelial cells on the luminal surface. Functionally, it plays an important role in enhancing the efflux of a number of substances from the brain into the vascular compartment, including beta amyloid [29]. Thus LRP and P-glycoprotein are felt to be the two principle proteins involved in mediating the efflux of beta amyloid from the brain into the vascular compartment, at the present time. The relative importance of these two proteins is not currently understood.

We have previously investigated the role of P-gp in AD lesion pathogenesis. In one study we observed a significant negative correlation between capillary P-gp expression and both neurofibrillary tangle [NFT] and SP lesions in AD, but not control, brain samples. In addition, we also observed significant positive correlations between P-gp capillary expression and both LRP and RAGE capillary expression in both AD and control brains [30]. We have also examined the regional variability of P-gp capillary expression between superior temporal cortex, hippocampal, and brain-stem samples in AD and control brains [31], noting particularly that there was a significant negative correlation between P-gp capillary expression and SP burden in the superior temporal cortex of AD brains, and brainstem levels were increased. The current study looking only at the ST cortex again emphasizes the significant negative correlation between P-gp expression and *β* amyloid plaque levels.

Another study has shown, in human AD autopsy brain, that in the hippocampus by immunofluorescence P-glycoprotein expression is reduced in hippocampal microvessels [29]. As well, in mouse models it has been shown that P-glycoprotein null mice have significant impairment of beta-40 and 42 peptide clearance, and, in addition, APP transgenic mice, when P-glycoprotein is inhibited, accumulate A-beta at an increased rate [32]. Finally in another transgenic mouse model of AD, restoring or increasing P-glycoprotein expression increased brain capillary A-beta transport activity and reduced brain A-beta levels [33]. All of these studies attest to the functional importance of P-glycoprotein in regulating beta-amyloid CNS levels and maintaining beta-amyloid clearance.

RAGE is known to facilitate amyloid influx into the cerebral interstitium from the vascular compartment. Under homeostatic conditions in non-Alzheimer brains it is likely that the efflux/influx ratio of A*β*_42_, the predominant amyloid peptide in senile plaques, produces a nonpathogenic balance. It is well documented that underexpression or impairment of either LRP or P-gp, both of which facilitate the transport of A*β* from the brain into the vascular compartment across the BBB endothelium, results in the pathogenic accumulation of A*β* peptide in the cerebral interstitium, thus facilitating senile plaque pathogenesis [5, 6, 9, 16]. Additionally, it has also been demonstrated that overexpression of RAGE results in an increased trans-endothelial transport of A*β* from the vascular compartment into the brain [16]. With regard to these transport activities it has been reported that P-gp may restrict RAGE activity [34]. The results of this study indicate both a significantly increased expression of RAGE in AD brains and a significant positive correlation between RAGE expression and SP burden. The fact that RAGE expression is increased is compatible with, therefore, an inferred mechanistic association with the observed increase in SP pathogenesis.

Based on the overview results of this study, the amyloid efflux transporter P-gp and both of the vasoactive proteins, eNOS and VEGF, are significantly and negatively correlated with A*β*_42_ senile plaques in the superior temporal cortex of Alzheimer brains. Both LRP and RAGE are significantly and positively correlated with A*β*_42_ senile plaque burden. This is interpreted as meaning that diminished expression and/or activity of P-gp, eNOS, and VEGF is at least linked, perhaps pathogenically, with senile plaque burden. Remarkably, in this study, we observed that LRP had a significant positive correlation with plaque burden, in contrast to an anticipated negative correlation in conjunction with that of the P-gp result. This would suggest that it is diminished amyloid efflux as a result of increased P-gp expression and activity, as well as an increased RAGE expression and activity, rather than LRP expression and activity which dominates the process by which A*β*_42_ senile plaques accumulate to neuropathologic levels.

What is unclear is whether there is a common regulatory mechanism that stimulates a response to the presence of amyloid by both the trans-endothelial transport proteins and the vasoactive proteins described here. Further research is required to investigate this possibility. Ultimately, both response pathways may require attention in the amelioration or elimination of Alzheimer lesion pathogenesis insofar as vascular dysfunctions are central to the pathogenesis of Alzheimer amyloid-associated lesions.

## 5. Conclusions

In the Alzheimer condition P-gp, VEGF, and eNOS positive capillaries were negatively correlated with SP burden; but LRP and RAGE were positively correlated with SP burden. These results strongly indicate the role of an altered BBB function in the pathogenesis of SPs in Alzheimer brains.

## Figures and Tables

**Table 1:** Summary of cases-mean ages and ranges for diagnostic data.

	Control	AD
Mean age	68	79
Braak and Braak stage	0	4–6
Cerad index	0-minimal	High

**Table 2:** Summary of raw data and significant difference results.

	A*β*_42_ plaques	LRP capillaries	RAGE capillaries	P-gp capillaries	eNOS capillaries	VEGF capillaries
Control	688	172	498	1279	73	921
AD	3776	196	648	920	104	674
Significant difference	*P* < 0.001	ns	*P* < 0.011	ns	ns	*P* < 0.035

**Table 3:** A*β*_42_ senile plaque and endothelial vasoactive proteins correlative data.

Endothelial cell-associated protein	LRP		P-gp		RAGE		eNOS		VEGF	
Condition	Control	AD	Control	AD	Control	AD	Control	AD	Control	AD
Correlation	Ns	Positive	Ns	Negative	Ns	Positive	Positive	Negative	Positive	Negative
Significance	Ns	*P* < 0.01	Ns	*P* < 0.01	Ns	*P* < 0.05	*P* < 0.05	*P* < 0.01	*P* < 0.01	*P* < 0.01
